# Better understanding of laboratory animal genetics will improve reproducibility

**DOI:** 10.1371/journal.pbio.3003464

**Published:** 2025-10-30

**Authors:** Guillaume Pavlovic, Sara Wells, Yann Hérault, Lydia Teboul

**Affiliations:** 1 PHENOMIN-Institut Clinique de la Souris, CELPHEDIA, CNRS, INSERM, Université de Strasbourg, Illkirch-Graffenstaden, France; 2 The Mary Lyon Centre at MRC Harwell, Harwell Campus, Didcot, United Kingdom; 3 Francis Crick Institute, London, United Kingdom; 4 Université de Strasbourg, CNRS, INSERM, IGBMC UMR 7104-UMR-S 1258, Illkirch-Graffenstaden, France

## Abstract

Incomplete characterization and inconsistent reporting of laboratory animal genetics undermine study quality and limit research reproducibility. This Perspective highlights the LAG-R guidelines and calls for collective action to address these critical gaps and enhance scientific integrity.

## Teaser

Inconsistent characterization and reporting of laboratory animal genetics undermine research quality and reproducibility. We need to recognize the value of genetic characterization, improve training for researchers, and implement rigorous reporting standards.

Laboratory animals are widely used in biomedical research, yet the potential experimental biases resulting from genetic factors are often underestimated. These complexities can arise from various sources: insufficient genomic validation [1]; unexpected functions of genetic alterations [2]; mid- and long-range effects of modification on the genetic locus [3]; unexpected patterns of gene expression modulation [4]; and the influence of genetic background [5], all of which can introduce confounding variables into *in vivo* experiments. Despite these potential sources of bias, such factors are often overlooked in the usual animal model characterization practices reported in publications.

So, what is missing? We would argue that there needs to be a fundamental recognition of the value and importance of thoroughly characterizing experimental models from the outset. In reality, however, the emphasis tends to be on obtaining experimental results quickly to maintain competitiveness and affordability. In the context of *in vivo* studies, a thorough understanding of experimental models requires training in genetics, initial investment in characterization within the relevant experimental context and comprehensive documentation of both genomic and functional validation. To improve the quality and reproducibility of laboratory animal studies, these three crucial aspects of the research process need to be prioritized.

First, we need to improve the level of expertise in animal genetics among research teams. At present, the expertise required to interpret the impact of animal genetics on experimental outcomes may not be present in every research team, and emphasis on specific aspects, such as genetic background or allele sequence, can also vary. Furthermore, laboratory animals are often just one of several experimental components, rather than the primary focus of a study. This can result in heterogenous validation and reporting of genetic backgrounds or of genetic alterations and their functional consequences. While a high level of expertise in animal genetics is not essential for all researchers who use laboratory animals, it is essential that all researchers adhere to reporting guidelines and use the applicable support frameworks available to them [6,7].

Second, we need more initial investment in model characterization within the relevant experimental context. The generation or acquisition of laboratory animal models often involves long timelines and the investment of significant resources. However, the pressure to publish or meet project deadlines frequently leads researchers to rush into acquisition of phenotypic experimental data without prior validation of the models, putting the entire investment at risk, as well as undermining both the reliability of the science and the ethical justification for using animals in research. Even when validation has been performed in other settings, the resulting information is rarely faithfully communicated during the transfer of animals between facilities or projects. This issue affects even widely used models [2,4]. Moreover, even well-characterized models may require additional validation when introduced into new experimental paradigms or environments [4]. Genomic verification of both the genetic background and the modified allele is essential and, in the case of engineered alleles, so too is the validation of the presumed functional outcome [2,4].

Third, extensive documentation of genomic and functional validation before, during, and after experimentation needs to become the norm, rather than the exception. An internal analysis by the PHENOMIN-ICS institute compared published descriptions of 27 recently reported animal models with their internal production records and found minor inaccuracies in nine cases and, more concerningly, major discrepancies in three models that may affect the reproducibility of the research. In addition, the sequence of the mutated allele was not provided for 20 of the 27 models (literature comparison to animal production data reviewed by GP/PHENOMIN-ICS). Similar observations of erroneous or incomplete reporting have been made by other animal facilities [8,9]. Strict compliance with established reporting guidelines would help to resolve such issues [6,7], as it would promote structured data acquisition throughout the experimental process, as well as ensuring comprehensive documentation at the publication stage.

Although the primary responsibility for how research is performed and reported lies with the authors, the reviewers and editors also have key roles in ensuring the quality and transparency of scientific publications. However, in an environment of increasingly multidisciplinary research, with increasingly varied and complex methods, reviewers and editors often lack expertise in every aspect of a given study, including the use and validation of laboratory animals. Despite this, they are still expected to assess the adequacy of model documentation during the review process. To facilitate this, we have participated in the creation of the LAG-R guidelines, which provide a list of fields for accurate genetic documentation to support non-specialists in animal genetics or genetic engineering [7]. They also serve as an off-the-shelf solution during the design and execution of experiments, listing the essential information to be reported in terms of genetic background, genetic alteration, and genetic validation. LAG-R belongs to a set of complementary guidelines including ARRIVE and PREPARE, which have checklists to support authors, reviewers, and editors in verifying that the description and validation of laboratory animals, as well as the related metadata, are reported in a thorough and appropriate fashion.

The issue of incomplete characterization of experimental models in science is not unique to laboratory animal research [7,10,11]. Standards of validation of experimental models and reporting guidelines remain to be adopted in many fields. One barrier to uptake lies with funding bodies, which often appear reluctant to allocate sufficient resources to validation work, as it is not perceived as innovative or cutting-edge science. A source of optimism is that the animal research community is being proactive in recognizing and addressing this issue [6,7,12]; however, these initiatives will have limited impact unless they are embraced across the entire scientific ecosystem — by funders, researchers, reviewers, and editors. Clarity regarding the description of animals in research and their intrinsic properties is the first step towards the responsible use of animals. It is now imperative that the broader scientific community recognizes the significance of the missing information and insufficient model validation, and takes collective action to fill that gap.
